# Prognostic Role of Immunonutritional Indices in Elderly Patients with HFpEF: Long-Term Follow-Up of the CONUT, PNI, and CALLy Scores

**DOI:** 10.3390/jcm15093245

**Published:** 2026-04-24

**Authors:** Andrea Sonaglioni, Chiara Lonati, Andrea Donzelli, Federico Napoli, Gian Luigi Nicolosi, Massimo Baravelli, Michele Lombardo, Sergio Harari

**Affiliations:** 1 Division of Cardiology, Istituto di Ricovero e Cura a Carattere Scientifico MultiMedica, 20123 Milan, Italy; massimo.baravelli@multimedica.it (M.B.); michele.lombardo@multimedica.it (M.L.); 2 Division of Internal Medicine, Istituto di Ricovero e Cura a Carattere Scientifico MultiMedica, 20123 Milan, Italy; chiara.lonati@multimedica.it (C.L.); andrea.donzelli3@studenti.unimi.it (A.D.); federico.napoli@multimedica.it (F.N.); sergio.harari@unimi.it (S.H.); 3 Division of Cardiology, Policlinico San Giorgio, 33170 Pordenone, Italy; gianluigi.nicolosi@gmail.com; 4 Department of Clinical Sciences and Community Health, Università Di Milano, 20122 Milan, Italy

**Keywords:** HFpEF, nutritional status, CONUT score, prognostic nutritional index, CALLy index, TAPSE/sPAP ratio, elderly patients, heart failure prognosis

## Abstract

**Background:** Malnutrition and systemic inflammation are increasingly recognized as important determinants of prognosis in patients with heart failure. Several immunonutritional indices, including the Prognostic Nutritional Index (PNI), the Controlling Nutritional Status (CONUT) score, and the C-reactive protein–albumin–lymphocyte (CALLy) index, have been proposed as markers of nutritional and inflammatory status. However, their prognostic value in elderly patients with heart failure with preserved ejection fraction (HFpEF) remains incompletely defined. This study aimed to evaluate the prognostic significance of these immunonutritional indices in elderly patients with HFpEF over a long-term follow-up period. **Methods:** This retrospective observational study included 200 elderly patients hospitalized with HFpEF (mean age 86.6 ± 6.5 years). Clinical, laboratory, and echocardiographic parameters were collected at admission. Nutritional status was assessed using PNI, CONUT score, and CALLy index. Patients were followed for mortality during long-term follow-up. Survival analyses were performed using Cox regression models, receiver operating characteristic (ROC) curves, and Kaplan–Meier analysis. Median follow-up was 3.8 years (IQR 2.1–5.9). **Results:** During follow-up, 123 patients (61.5%) died, while 77 patients (38.5%) were alive at the end of observation. In univariate analysis, PNI, CONUT score, left ventricular ejection fraction (LVEF), and the tricuspid annular plane systolic excursion to systolic pulmonary artery pressure (TAPSE/sPAP) ratio were significantly associated with mortality. In multivariate analysis, the CONUT score, LVEF, and the TAPSE/sPAP ratio remained independent predictors of mortality. ROC analysis showed strong prognostic performance for the TAPSE/sPAP ratio (AUC 0.932), CONUT score (AUC 0.925), and LVEF (AUC 0.897). Optimal cut-off values for mortality prediction were CONUT ≥ 6, LVEF ≥ 65%, and TAPSE/sPAP ≤ 0.55 mm/mmHg. Kaplan–Meier analysis confirmed significantly reduced survival among patients with higher CONUT scores, higher LVEF, and an impaired TAPSE/sPAP ratio. **Conclusions:** In elderly patients with HFpEF, nutritional status and cardiopulmonary functional parameters are important determinants of long-term prognosis. The CONUT score emerged as the most informative immunonutritional index, while echocardiographic parameters reflecting ventricular function and right ventricular–pulmonary arterial coupling provided additional prognostic information. Integrating nutritional assessment with echocardiographic evaluation may improve risk stratification in elderly patients with HFpEF.

## 1. Introduction

Heart failure (HF) represents one of the leading causes of morbidity and mortality worldwide and constitutes a major public health burden, particularly in aging populations [1,2,3]. The progressive aging of Western societies has resulted in a growing number of elderly patients affected by HF, with a substantial proportion of hospitalizations and deaths occurring in individuals over 80 years of age [4,5,6,7]. In this setting, heart failure with preserved ejection fraction (HFpEF) has become the predominant HF phenotype among older patients, accounting for more than half of HF cases in individuals of advanced age [8,9,10,11].

HFpEF is a complex and heterogeneous syndrome characterized by multiple comorbidities, systemic inflammation, and age-related physiological changes [12,13,14]. Unlike heart failure with reduced ejection fraction, HFpEF is strongly influenced by non-cardiac factors such as renal dysfunction, frailty, metabolic disorders, and systemic inflammatory conditions [15,16,17,18]. In very old patients, the clinical course of HFpEF is often determined not only by cardiac dysfunction but also by extra-cardiac factors including infections, nutritional status, and immune dysregulation [19,20,21,22,23,24].

Malnutrition has emerged as an important determinant of prognosis in patients with HF. Nutritional impairment is highly prevalent in HF and has been associated with worse clinical outcomes, including increased mortality and hospitalizations [25,26,27,28]. The mechanisms underlying malnutrition in HF are multifactorial and include chronic inflammation, neurohormonal activation, reduced appetite, gastrointestinal congestion, and metabolic abnormalities [29,30,31]. These processes are particularly relevant in elderly individuals, in whom malnutrition frequently coexists with frailty and immunological impairment [32,33,34].

For this reason, increasing attention has been directed toward the assessment of nutritional status using simple and reproducible indices. Among these, the Prognostic Nutritional Index (PNI), based on serum albumin levels and lymphocyte count, reflects both nutritional and immune status and has been investigated as a prognostic marker in cardiovascular diseases and HF populations [35,36,37,38]. Similarly, the Controlling Nutritional Status (CONUT) score, which incorporates serum albumin, total cholesterol levels, and lymphocyte count, was initially developed as a screening tool for hospital malnutrition and has subsequently been validated as a prognostic marker in several cardiovascular conditions, including acute and chronic HF [39,40,41,42,43,44,45,46,47,48,49]. More recently, the C-reactive protein–albumin–lymphocyte (CALLy) index has been proposed as a composite marker integrating nutritional and inflammatory parameters. By combining C-reactive protein, albumin concentration, and lymphocyte count, this index aims to capture the interaction between systemic inflammation and nutritional impairment [50,51,52,53,54]. However, evidence regarding its prognostic role in HF populations remains limited.

In elderly patients with HF, the interplay between malnutrition, inflammation, and immune dysfunction plays a key role in prognosis. Aging-related immunosenescence predisposes to infections and systemic inflammation [55], which may exacerbate catabolic processes and disease progression; infections, particularly respiratory, are frequent causes of hospitalization and death in this population [56,57].

Despite the recognized prognostic importance of nutritional status in HF, data on immunonutritional indices in very elderly HFpEF patients remain limited. This population is characterized by a high burden of comorbidities, frailty, and functional impairment, all of which may influence outcomes [58].

Our group has previously investigated clinical features of elderly HF patients, including HF with supranormal ejection fraction and risk scores [59,60,61], but the prognostic role of nutritional and immunological markers has not been systematically assessed.

Therefore, this study aimed to evaluate the prognostic value of PNI, CONUT, and CALLy indices in very elderly HFpEF patients, assessing their association with all-cause mortality and their ability to improve risk stratification.

## 2. Materials and Methods

### 2.1. Study Design and Population

The present study was designed as a retrospective observational cohort study aimed at evaluating the prognostic role of immunonutritional indices in elderly patients with HFpEF. The study population consisted of consecutive patients admitted to the Internal Medicine and Cardiology Units of IRCCS MultiMedica—San Giuseppe Hospital (Milan, Italy) for HF between January 2020 and December 2020.

A total of 200 patients were included in the study. HFpEF was defined according to current guideline-based criteria [62] and consistently adjudicated using a comprehensive multiparametric approach, in line with previous studies conducted by our group [59,60,61]. Specifically, the diagnosis of HFpEF required the simultaneous presence of: (1) typical symptoms (e.g., dyspnea, fatigue, reduced exercise tolerance) and/or signs (e.g., pulmonary rales, peripheral edema, elevated jugular venous pressure) of heart failure; (2) preserved left ventricular ejection fraction (LVEF) (≥50%) assessed by transthoracic echocardiography performed during hospitalization; (3) elevated levels of natriuretic peptides, with N-terminal pro–brain natriuretic peptide (NT-proBNP) values above age-adjusted diagnostic thresholds (≥125–450 pg/mL depending on clinical context); and (4) objective evidence of structural and/or functional cardiac abnormalities, including left ventricular hypertrophy, left atrial enlargement, and/or diastolic dysfunction as assessed by Doppler parameters (E/A ratio and E/e′ ratio), reflecting increased left ventricular filling pressures.

Given the advanced age and high comorbidity burden of the study population, particular attention was paid to differentiating HFpEF from alternative or concomitant causes of acute dyspnea and clinical deterioration. To this end, all cases were systematically reviewed by experienced clinicians (internal medicine and cardiology specialists) who integrated clinical presentation, laboratory findings, imaging data, and response to therapy. Competing conditions such as acute pulmonary infections, chronic obstructive pulmonary disease exacerbations, severe anemia, renal failure, and systemic inflammatory states were carefully evaluated. HFpEF decompensation was adjudicated only when signs and symptoms of congestion and/or elevated filling pressures were considered predominant and not fully explained by non-cardiac conditions.

In addition, consistent with our prior studies [59,60,61], all patients underwent comprehensive clinical, laboratory, chest X-ray, and echocardiographic assessment within the first 24–48 h of admission, allowing for a standardized diagnostic process. When multiple potential causes of hospitalization were present—as frequently observed in this elderly population—HFpEF was considered the primary diagnosis only in the presence of concordant clinical, biochemical (including natriuretic peptides), and echocardiographic evidence of cardiac decompensation.

All patients were followed longitudinally to assess long-term outcomes, with follow-up extending until February 2026.

In contrast to our previous studies, which explored various clinical aspects of elderly patients with HF [59,60,61], the present study addresses a distinct research question, with different objectives, variables of interest, and analytical focus. Specifically, one prior study evaluated the prognostic role of supra-normal LVEF (≥65%) and its association with mortality and rehospitalization in elderly HFpEF patients, primarily focusing on echocardiographic phenotypes and ventricular functional patterns [59]. Another analysis assessed the prevalence and prognostic implications of different echocardiographic and hemodynamic HF phenotypes in hospitalized elderly patients, without including nutritional or inflammatory parameters [60]. In addition, we previously examined the prognostic value of clinical risk scores, including the CHA_2_DS_2_-VASc score, with particular emphasis on comorbidity burden and thromboembolic risk stratification [61].

Importantly, none of these studies included an evaluation of immunonutritional indices or explored the interplay between nutritional status, systemic inflammation, and immune function in determining long-term outcomes. Therefore, despite partial cohort overlap, the present study addresses a distinct and novel research question, focusing on the prognostic significance of immunonutritional indices (PNI, CONUT, and CALLy) and their integration with echocardiographic parameters in very elderly HFpEF patients.

### 2.2. Data Collection

Clinical, laboratory, and instrumental data were retrospectively obtained from electronic medical records at the time of hospital admission. Demographic information including age and sex was collected together with data regarding cardiovascular risk factors such as hypertension, diabetes mellitus, dyslipidemia, smoking status, and obesity.

Information on non-cardiovascular comorbidities including chronic kidney disease, chronic obstructive pulmonary disease, hypothyroidism, and cognitive impairment was also recorded. In addition, cardiovascular comorbidities such as coronary artery disease, previous transient ischemic attack or stroke, and peripheral artery disease were documented.

Vital signs measured at admission were recorded, including systolic and diastolic blood pressure, heart rate, and body temperature. Information regarding the primary cause of hospital admission, pharmacological therapies administered during hospitalization, and length of hospital stay was also retrieved from the medical records.

### 2.3. Laboratory Assessment

Blood samples were collected at hospital admission as part of routine clinical evaluation. Laboratory parameters included hemoglobin levels, white blood cell count, neutrophil count, lymphocyte count, platelet count, serum glucose, serum iron, serum creatinine, estimated glomerular filtration rate (eGFR) [63], sodium and potassium levels, serum calcium, total bilirubin, albumin concentration, uric acid levels, and lipid profile including total cholesterol, high-density lipoprotein cholesterol (HDL cholesterol), low-density lipoprotein cholesterol (LDL cholesterol), and triglycerides.

Additional laboratory measurements included thyroid-stimulating hormone, C-reactive protein, NT-proBNP, and high-sensitivity cardiac troponin.

### 2.4. Nutritional Risk Scores

Three immunonutritional indices were calculated for each patient using laboratory parameters obtained at hospital admission.

The PNI was calculated using a formula combining serum albumin concentration and total lymphocyte count. Specifically, PNI was derived using the equation PNI = 10 × serum albumin (g/dL) + 0.005 × total lymphocyte count (cells/mm^3^) [35,36,37,38].

The CONUT score was calculated using three laboratory parameters: serum albumin concentration, total cholesterol level, and total lymphocyte count. According to the original scoring system proposed by Ignacio de Ulíbarri and colleagues [64], each parameter contributes to a weighted score reflecting the degree of nutritional impairment. Serum albumin levels ≥ 3.5 g/dL correspond to 0 points, levels between 3.0 and 3.49 g/dL correspond to 2 points, levels between 2.5 and 2.99 g/dL correspond to 4 points, and levels < 2.5 g/dL correspond to 6 points. For total lymphocyte count, values ≥ 1600 cells/mm^3^ correspond to 0 points, values between 1200 and 1599 cells/mm^3^ correspond to 1 point, values between 800 and 1199 cells/mm^3^ correspond to 2 points, and values < 800 cells/mm^3^ correspond to 3 points. Regarding total cholesterol concentration, values ≥ 180 mg/dL correspond to 0 points, values between 140 and 179 mg/dL correspond to 1 point, values between 100 and 139 mg/dL correspond to 2 points, and values < 100 mg/dL correspond to 3 points. The final CONUT score is obtained by summing the points assigned to each parameter, with higher scores indicating more severe nutritional impairment and immune dysfunction [42,44,46].

The CALLy index was calculated as a composite biomarker integrating inflammatory and nutritional parameters. The CALLy index was derived using the following formula: CALLy index = (serum albumin [g/dL] × lymphocyte count [/mm^3^])/C-reactive protein (mg/dL) [50,51,52,53,54].

To facilitate the interpretation of the immunonutritional indices used in the present study, the commonly reported reference ranges and risk categories for the PNI, the CONUT score, and the CALLy index are summarized in Table 1.

### 2.5. Echocardiographic Assessment

All patients underwent transthoracic echocardiography during hospitalization as part of routine clinical evaluation. Echocardiographic examinations were performed within 48 h of hospital admission using standard ultrasound equipment.

All echocardiographic studies were performed by a single experienced operator, in order to minimize inter-observer variability. Measurements were obtained according to current recommendations for cardiac chamber quantification and echocardiographic assessment [65,66].

Standard two-dimensional and Doppler parameters were recorded, including interventricular septal thickness, posterior wall thickness, left ventricular (LV) end-diastolic diameter, relative wall thickness (RWT), LV end-diastolic and end-systolic volumes, and LVEF. Doppler measurements included the E/A ratio and E/e’ ratio for the evaluation of LV diastolic function.

Left atrial dimensions and volume were measured, and the presence and severity of valvular abnormalities were assessed [67]. Right ventricular (RV) function was evaluated by measuring tricuspid annular plane systolic excursion (TAPSE), while systolic pulmonary artery pressure (sPAP) was estimated using Doppler-derived measurements [68].

The ratio between TAPSE and sPAP was calculated as a noninvasive index of RV–pulmonary arterial (PA) coupling [69].

### 2.6. Follow-Up and Outcome Definition

Patients were followed longitudinally from the time of hospital admission until 2026. Follow-up data were obtained through hospital records and clinical follow-up information.

The primary outcome of the study was all-cause mortality during the follow-up period. Based on survival status at the end of follow-up, patients were categorized into two groups: those who were alive and those who had died during the observation period.

### 2.7. Statistical Analysis

Continuous variables were expressed as mean ± standard deviation or as median and interquartile range depending on the distribution of the data. Categorical variables were presented as absolute numbers and percentages.

Comparisons between groups were performed using Student’s *t*-test or the Mann–Whitney U test for continuous variables and the chi-square test for categorical variables.

Univariate Cox proportional hazards regression analysis was used to identify variables associated with mortality. A predefined set of clinically relevant variables, including demographic characteristics, comorbidities, laboratory parameters, and echocardiographic indices, was initially considered based on prior evidence and pathophysiological plausibility in HFpEF populations. To ensure model parsimony and avoid overfitting given the sample size and number of events, only variables showing significant associations in univariate analysis were subsequently entered into multivariate Cox regression models. In addition, variables with potential collinearity or overlapping biological meaning (particularly among immunonutritional indices) were carefully evaluated, and only the most informative parameters were retained in the final model.

Receiver operating characteristic (ROC) curve analysis was performed to evaluate the predictive performance of nutritional scores and echocardiographic parameters. The area under the curve (AUC) was calculated for each variable, and optimal cut-off values were determined using the Youden index.

Kaplan–Meier survival curves were constructed to evaluate differences in survival between groups, and statistical significance was assessed using the log-rank test.

A *p*-value lower than 0.05 was considered statistically significant. All statistical analyses were performed using SPSS software version 28 (SPSS Inc., Chicago, IL, USA).

### 2.8. Ethical Approval

The study was conducted in accordance with the principles of the Declaration of Helsinki. The study protocol was reviewed and approved by the local Ethics Committee (reference number 464.2021). Because of the study’s retrospective design and the use of anonymized clinical data, the requirement for written informed consent was waived in accordance with national regulations.

### 2.9. Use of Artificial Intelligence Tools

Artificial intelligence (AI) assistance was used exclusively for linguistic revision and improvement of language clarity during manuscript preparation. ChatGPT (OpenAI, San Francisco, CA, USA) was employed only to help refine grammar, spelling, and readability of the text. AI tools were not involved in literature screening, data collection, statistical analyses, or interpretation of the study findings. The authors retain full responsibility for the scientific content and conclusions presented in this work.

## 3. Results

### 3.1. Baseline Characteristics

The study population consisted of 200 elderly patients hospitalized for HFpEF. The demographic and clinical characteristics of the study population are summarized in Table 2.

The overall cohort was characterized by advanced age and a predominance of female patients. The prevalence of traditional cardiovascular risk factors, including hypertension, diabetes mellitus, dyslipidemia, smoking, and obesity, was similar between patients who survived and those who died during follow-up.

Among non-cardiovascular comorbidities, chronic kidney disease and cognitive impairment were more frequently observed among patients who died during follow-up. In contrast, other comorbid conditions such as chronic obstructive pulmonary disease and hypothyroidism showed comparable distributions between the two groups.

Regarding clinical presentation at admission, some differences were observed in vital parameters between patients who survived and those who died during follow-up. In particular, patients who died tended to present with lower systolic blood pressure and higher heart rate at admission, suggesting a more compromised hemodynamic profile.

Body temperature at admission also differed between groups, with fever being more frequently documented among patients who died during follow-up. In contrast, symptoms related to congestion, including dyspnea and peripheral edema, were similarly distributed between the two groups.

### 3.2. Laboratory Findings and Nutritional Scores

Laboratory parameters and immunonutritional indices at hospital admission are reported in Table 3.

Markers of systemic inflammation and hematologic parameters differed between the two groups, with higher inflammatory activity observed among patients who died during follow-up. In particular, patients who died exhibited higher white blood cell and neutrophil counts, together with lower lymphocyte levels, indicating a shift toward a more pronounced inflammatory and immune-dysregulated profile. Differences were also observed in renal function parameters and selected metabolic variables. Specifically, the deceased group showed higher creatinine levels and lower eGFR, consistent with more advanced renal impairment, as well as lower serum iron concentrations.

Parameters reflecting nutritional status showed significant differences between groups. In particular, serum albumin and lipid profile parameters were lower among patients who died during follow-up. This pattern was characterized by reduced albumin levels and lower total, HDL, and LDL cholesterol concentrations, suggesting a state of protein–calorie malnutrition and reduced metabolic reserve in non-survivors.

Similarly, biomarkers related to HF severity, including natriuretic peptides, were higher among patients who died compared with those who survived. In addition, inflammatory burden was further supported by higher CRP levels, while NT-proBNP concentrations were markedly elevated, reflecting greater hemodynamic stress and disease severity in this group.

The distribution of the three immunonutritional indices evaluated in the present study—the PNI, the CONUT score, and the CALLy index—is also shown in Table 3. Significant differences between groups were observed for all three indices, with lower PNI and CALLy index values and higher CONUT scores among patients who died during follow-up. Overall, non-survivors displayed a consistent pattern of worse immunonutritional status, characterized by lower PNI, markedly reduced CALLy index values, and substantially higher CONUT scores, indicating the coexistence of malnutrition, systemic inflammation, and immune dysfunction.

### 3.3. Instrumental Findings

Chest radiography, electrocardiographic, and echocardiographic findings at hospital admission are shown in Table 4.

Regarding chest radiography, differences were observed in the distribution of radiographic patterns between groups. In particular, radiographic evidence of pulmonary infection was more frequently observed among patients who died during follow-up, whereas a normal radiographic pattern was more common among survivors. Pulmonary congestion was observed with comparable frequency in the two groups.

Electrocardiographic findings were largely similar between groups in terms of rhythm and conduction abnormalities. The prevalence of sinus rhythm, atrial fibrillation, and most conduction patterns did not differ significantly. However, right bundle branch block was more frequent among patients who died during follow-up, suggesting greater conduction system involvement or right ventricular dysfunction, whereas other abnormalities were comparable between groups.

Echocardiographic parameters describing cardiac structure showed some differences between patients who survived and those who died during follow-up. In particular, patients who died were characterized by smaller LV cavity dimensions and a more pronounced pattern of concentric remodeling. This was reflected by higher indices of wall thickness and RWT, suggesting a more hypertrophic ventricular geometry.

In addition, LV systolic function appeared more hyperdynamic among patients who died during follow-up, with higher ejection fraction values compared with survivors.

Differences were also observed in parameters reflecting RV function and pulmonary hemodynamics. In particular, patients who died showed lower TAPSE and higher pulmonary pressures, resulting in markedly lower values of the TAPSE/sPAP ratio.

### 3.4. Clinical Characteristics, Causes of Admission, and In-Hospital Treatment

Clinical characteristics at admission, etiological factors, causes leading to hospitalization, and treatments administered during hospitalization are reported in Table 5.

With respect to functional status at admission, differences were observed between patients who died during follow-up and those who survived. In particular, NYHA class IV was more frequently observed among patients who died, whereas NYHA class III was more common among survivors.

Differences were also found in the underlying etiology of HF. Pulmonary hypertension was significantly more frequent among patients who died, while hypertensive heart disease was more commonly observed among survivors. Other etiologies, including coronary artery disease and valvular heart disease, showed comparable distributions between the two groups.

The clinical conditions leading to hospital admission also differed between groups. Acute respiratory conditions represented the most frequent cause of admission among patients who died, whereas congestive HF was more commonly observed among survivors. In addition, the presence of two or more concurrent causes of admission was more frequent among patients who died during follow-up.

Regarding in-hospital treatment, some differences in pharmacological therapy were observed. ACE inhibitors or angiotensin receptor blockers were more frequently prescribed among survivors, while statins, antibiotic therapy, and oxygen therapy were more commonly administered to patients who died during follow-up.

Finally, length of hospital stay was longer among patients who died during follow-up compared with those who survived.

### 3.5. Follow-Up and Clinical Events

Follow-up data were available for the entire study population. The median duration of follow-up was 3.8 years (interquartile range 2.1–5.9 years).

During the observation period, a substantial proportion of patients died, while the remaining individuals were alive at the end of follow-up but frequently experienced non-fatal clinical events. The distribution of mortality and the types of clinical events observed during follow-up are summarized in Table 6.

Respiratory and infectious conditions represented the most frequent causes of death in this cohort, followed by cardiovascular causes and other non-cardiovascular medical conditions. A proportion of deaths was also associated with progressive clinical deterioration and frailty-related complications.

Among patients who survived during follow-up, several experienced non-fatal events require medical evaluation, emergency department visits, or hospital admissions. These events were most commonly related to respiratory conditions, although cardiovascular complications, infectious diseases, metabolic disturbances, and trauma-related events were also observed.

Early mortality during the index hospitalization was relatively uncommon, occurring in 11 patients (5.5% of the study population), while the vast majority of deaths occurred during the post-discharge follow-up period.

### 3.6. Univariate and Multivariate Cox Regression Analysis

Univariate Cox regression analysis was performed to identify clinical, laboratory, and echocardiographic variables associated with mortality during follow-up. The results of both univariate and multivariate analyses are reported in Table 7.

In univariate analysis, several variables showed significant associations with mortality, including the PNI, the CONUT score, LVEF, and the TAPSE/sPAP ratio.

Variables that reached statistical significance in univariate analysis were subsequently entered into a multivariate Cox regression model. In the multivariate analysis, CONUT score, LVEF, and TAPSE/sPAP ratio remained independently associated with mortality. In contrast, the association between PNI and mortality was no longer significant after adjustment for other variables.

### 3.7. ROC Analysis

**Figure 1:**
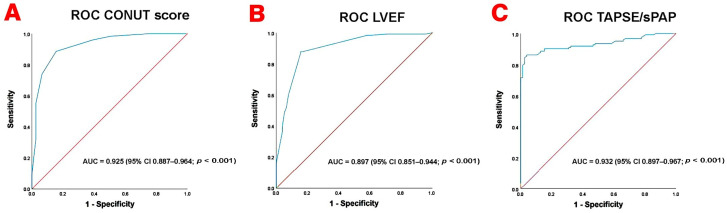
Receiver operating characteristic (ROC) curves evaluating the prognostic performance of selected nutritional and echocardiographic parameters for the prediction of mortality during follow-up in patients with heart failure with preserved ejection fraction (HFpEF). (**A**) Controlling Nutritional Status (CONUT) score. (**B**) Left ventricular ejection fraction (LVEF). (**C**) Tricuspid annular plane systolic excursion-to-systolic pulmonary artery pressure ratio (TAPSE/sPAP). The area under the curve (AUC) values with 95% confidence intervals are reported within each panel.

ROC curve analysis was performed to evaluate the ability of selected nutritional and echocardiographic parameters to predict mortality during follow-up. The ROC curves are shown in Figure 1.

The TAPSE/sPAP ratio demonstrated the highest discriminative performance, with an AUC of 0.932 (95% CI 0.897–0.967; *p* < 0.001). The CONUT score also showed excellent predictive ability, with an AUC of 0.925 (95% CI 0.887–0.964; *p* < 0.001). In addition, LVEF demonstrated good prognostic performance, with an AUC of 0.897 (95% CI 0.851–0.944; *p* < 0.001).

Overall, these results indicate that both nutritional statuses assessed by the CONUT score and cardiopulmonary functional parameters such as TAPSE/sPAP ratio and LVEF showed strong discriminative capacity for predicting mortality in this cohort.

### 3.8. Kaplan–Meier Survival Analysis

**Figure 2:**
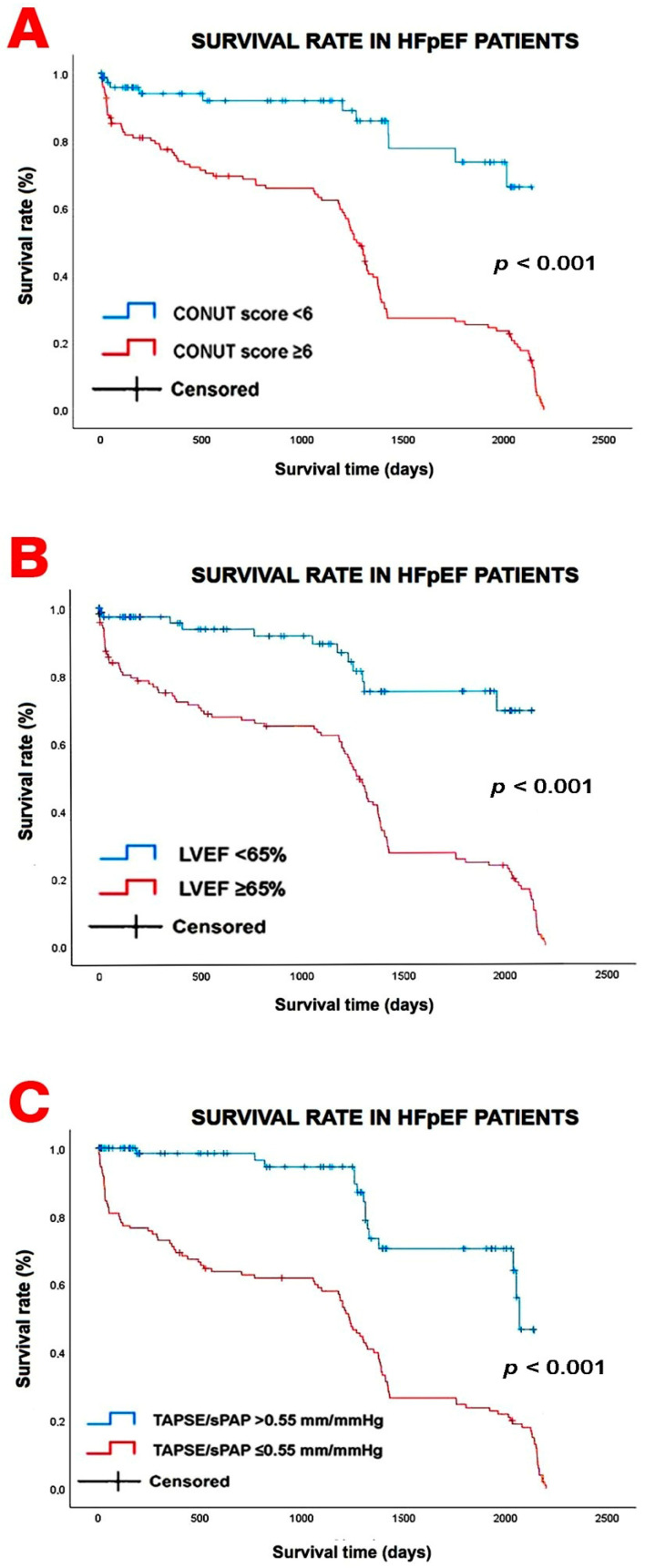
Kaplan–Meier survival curves illustrating differences in survival according to (**A**) CONUT score (<6 vs. ≥6), (**B**) left ventricular ejection fraction (LVEF < 65% vs. ≥65%), and (**C**) tricuspid annular plane systolic excursion-to-systolic pulmonary artery pressure ratio (TAPSE/sPAP > 0.55 vs. ≤0.55 mm/mmHg). Survival differences between groups were assessed using the log-rank test.

Kaplan–Meier survival curves were constructed to evaluate differences in survival according to the main prognostic variables identified in the regression analyses. The survival curves are presented in Figure 2.

Using the optimal thresholds derived from ROC curve analysis, patients were stratified according to CONUT score ≥ 6, LVEF ≥ 65%, and TAPSE/sPAP ratio ≤ 0.55 mm/mmHg. The CONUT cut-off of 6 showed a sensitivity of 88.6% and a specificity of 84.4% for predicting mortality. The optimal LVEF threshold was 65%, corresponding to a sensitivity of 87.8% and a specificity of 83.1%. For the TAPSE/sPAP ratio, the best prognostic cut-off was 0.55 mm/mmHg, with a sensitivity of 85.4% and a specificity of 96.1%.

Patients with CONUT scores ≥6 showed significantly lower survival probabilities during follow-up compared with patients with lower scores. Similarly, a TAPSE/sPAP ratio ≤ 0.55 mm/mmHg was associated with markedly worse survival. Survival differences were also observed according to LVEF values: patients with supranormal LVEF (≥65%) exhibited lower survival rates compared with those with normal LVEF (<65%), suggesting that very high ejection fraction values are associated with poorer outcomes.

The differences between groups were statistically significant according to the log-rank test, confirming the prognostic relevance of these parameters.

## 4. Discussion

### 4.1. Principal Findings

The present study evaluated the prognostic relevance of three immunonutritional indices—PNI, CONUT score, and CALLy index—in a cohort of elderly patients hospitalized with HFpEF and followed over a prolonged observational period.

Several relevant findings emerge from our analysis and contribute to a better understanding of the determinants of prognosis in this complex and understudied population.

First, our data highlight the important role of nutritional status in the prognostic stratification of elderly patients with HFpEF. Among the indices analyzed, the CONUT score showed the strongest and most consistent association with mortality, maintaining its prognostic significance even after adjustment for other clinical and echocardiographic variables. In contrast, the prognostic value of PNI was attenuated after multivariable adjustment, while the CALLy index did not emerge as an independent predictor of outcome. These results indicate that composite immunonutritional indices may provide incremental prognostic information beyond conventional clinical markers.

It should be noted, however, that these three indices are not entirely independent constructs. All of them share partially overlapping components—particularly serum albumin and lymphocyte count—and therefore reflect closely related biological domains linking nutritional status, immune competence, and systemic inflammation. The stronger prognostic performance of the CONUT score observed in our study may therefore be explained by its broader multidimensional structure, as it incorporates an additional parameter (total cholesterol) reflecting caloric depletion and metabolic reserve. Accordingly, CONUT may offer a more comprehensive representation of the biological vulnerability typical of very elderly HFpEF patients.

Second, echocardiographic parameters reflecting ventricular function and cardiopulmonary interaction also played a central role in risk stratification. In particular, markers related to RV performance and RV–PA coupling emerged as powerful predictors of outcome. The TAPSE/sPAP ratio showed a strong association with survival, highlighting the importance of right ventricular function and pulmonary hemodynamics in determining prognosis in patients with HFpEF. In addition, LV systolic function, although preserved by definition in this population, showed a significant relationship with mortality when considered across its physiological range.

Consistent with these findings, the comparison between patients who died and those who survived during follow-up revealed distinct clinical and functional characteristics associated with adverse outcomes. Patients who died tended to present at admission with a more compromised hemodynamic profile, characterized by lower systolic blood pressure and higher heart rate. They also showed a higher prevalence of systemic inflammatory activation and markers suggestive of impaired nutritional status.

From a structural and functional standpoint, the echocardiographic profile of patients who died was characterized by features consistent with a more advanced HFpEF phenotype. These patients frequently exhibited smaller LV cavity dimensions together with increased wall thickness, suggesting a pattern of concentric remodeling or hypertrophy. In addition, LV systolic function tended to be more hyperdynamic in this group. At the same time, indices reflecting RV function and pulmonary circulation were markedly impaired among patients who died, particularly the TAPSE/sPAP ratio, indicating a significant alteration in RV–PA coupling.

Finally, the long-term follow-up of this cohort confirmed the high burden of mortality and clinical instability typical of very elderly patients with HFpEF. Deaths occurred progressively during follow-up and were often preceded by episodes of clinical deterioration, with respiratory and infectious conditions representing frequent causes of clinical events. Overall, these observations highlight the interplay between nutritional impairment, systemic inflammation, and cardiopulmonary dysfunction in shaping prognosis in this population.

### 4.2. Interpretation of Findings and Comparison with Previous Studies

The findings of the present study contribute to the growing body of evidence supporting the prognostic relevance of nutritional status in patients with HF. In our cohort of elderly patients with HFpEF, nutritional impairment assessed through composite immunonutritional indices was strongly associated with long-term outcomes.

Among the indices evaluated, the CONUT score showed the most robust prognostic performance, remaining independently associated with mortality after adjustment for clinical and echocardiographic variables. These results are consistent with previous studies demonstrating that malnutrition and immune dysfunction represent important determinants of adverse outcomes in patients with HF. Several investigations have shown that the CONUT score, which integrates serum albumin levels, lymphocyte count, and cholesterol concentration, is associated with mortality and hospitalization risk in patients with both reduced and preserved ejection fraction [42,44,46]. In addition, previous reports have highlighted that CONUT may provide incremental prognostic information beyond traditional risk markers in HF populations [43,45].

Similarly, the PNI has been investigated as a marker of nutritional and inflammatory status in cardiovascular disease. Several studies have demonstrated that lower PNI values are associated with increased mortality and worse clinical outcomes in patients with HF and other cardiovascular conditions [35,36,37,38]. However, the independent prognostic value of PNI appears to be less consistent across studies, particularly after adjustment for clinical and laboratory variables, which is consistent with the attenuation observed in our multivariate analysis.

The CALLy index, originally proposed as an inflammatory–nutritional biomarker in oncologic and systemic diseases, has more recently been explored in cardiovascular populations. Although previous studies have suggested that this index may reflect the interaction between inflammation and nutritional status [50], its prognostic value in HF remains less clearly established. In the present study, the CALLy index showed a weaker association with mortality compared with CONUT and did not remain significant after multivariable adjustment.

These findings are in line with previous literature suggesting that different immunonutritional indices may capture partially distinct aspects of the complex interaction between malnutrition, systemic inflammation, and immune dysfunction in HF populations [70,71,72].

Beyond nutritional status, our findings also emphasize the importance of cardiopulmonary functional parameters in determining prognosis in HFpEF. In particular, the TAPSE/sPAP ratio emerged as a strong predictor of mortality, highlighting the critical role of RV–PA coupling. Previous studies have demonstrated that impaired RV function and increased pulmonary pressures are key determinants of adverse outcomes in HFpEF [73,74,75,76].

Interestingly, our study also identified LVEF as a variable associated with outcome despite the preserved ejection fraction typical of this population. This observation likely reflects the complex relationship between ventricular geometry, myocardial mechanics, and systemic conditions in elderly patients with HFpEF. In particular, patients who died in our cohort frequently exhibited concentric remodeling and relatively hyperdynamic ventricular function, a phenotype previously reported in HF populations [77,78,79,80].

A key aspect in the interpretation of these findings relates to the advanced age and high comorbidity burden of the study population. In our cohort, non-survivors more frequently presented with clinical features suggestive of acute systemic illness, including fever, pneumonia, need for oxygen therapy, and antibiotic treatment, as well as a higher prevalence of respiratory causes of death. These observations indicate that mortality was likely influenced not only by HFpEF-related mechanisms but also by acute non-cardiac conditions, particularly infectious and respiratory diseases.

In this context, immunonutritional indices such as the CONUT score may reflect a broader biological vulnerability, capturing the combined effects of malnutrition, systemic inflammation, frailty, and acute illness severity, rather than being exclusively specific to cardiac dysfunction. This is consistent with the components of the CONUT score (albumin, lymphocyte count, and cholesterol), which are known to be significantly influenced by inflammatory states and acute infections.

Although multivariable models were adjusted for several clinical and echocardiographic variables, residual confounding related to acute illness severity cannot be entirely excluded. Therefore, the prognostic value of CONUT and related indices in this population should be interpreted as reflecting overall clinical vulnerability and systemic disease burden, rather than disease-specific mechanisms alone.

From a clinical perspective, however, this broader prognostic signal remains highly relevant, as elderly patients with HFpEF are typically characterized by multimorbidity and competing risks, and outcomes are often driven by the complex interplay between cardiac and extra-cardiac conditions.

### 4.3. Clinical Implications

The findings of the present study have several potential implications for the clinical management of elderly patients with HFpEF.

First, our results indicate that simple and widely available immunonutritional indices may provide meaningful prognostic information in routine clinical practice. Among these, the CONUT score emerged as the most informative marker in our cohort. Because it can be easily calculated from routinely available laboratory parameters, this score may represent a practical tool for identifying elderly HFpEF patients at increased risk of adverse outcomes. Early recognition of impaired nutritional status may allow clinicians to implement targeted strategies aimed at improving nutritional balance, reducing systemic inflammatory burden, and optimizing overall clinical management.

Second, the strong prognostic value observed for the TAPSE/sPAP ratio underscores the importance of systematically assessing RV function and pulmonary hemodynamics in patients with HFpEF. Incorporation of this parameter into routine echocardiographic evaluation may improve risk stratification and facilitate the identification of patients with more advanced cardiopulmonary impairment.

Third, the combined evaluation of nutritional markers and echocardiographic parameters may allow a more comprehensive assessment of patient vulnerability. Elderly individuals with HFpEF often present with a complex interaction between comorbidities, systemic inflammation, and cardiovascular dysfunction. In this population, malnutrition, chronic inflammatory activation, and acute infectious conditions frequently coexist and may contribute to a state of heightened biological vulnerability. Systemic inflammation may promote endothelial dysfunction, microvascular impairment, and myocardial remodeling, whereas malnutrition may exacerbate frailty, impair immune response, and reduce physiological reserve [81].

Importantly, the identification of specific cut-off values derived from ROC curve analysis may facilitate the translation of these findings into clinical practice. In our study, a CONUT score ≥ 6, a TAPSE/sPAP ratio ≤ 0.55 mm/mmHg, and a supranormal LVEF ≥ 65% were each associated with a significantly increased risk of mortality. When considered together, these parameters may allow a simple and clinically applicable stratification of patient risk, whereby the accumulation of abnormal findings reflects a progressively higher degree of vulnerability. Patients presenting with multiple high-risk features may therefore require closer clinical monitoring and a more intensive, multidisciplinary management approach, including optimization of HF therapy and targeted nutritional interventions. In practical terms, the presence of ≥2 abnormal parameters (CONUT ≥ 6, TAPSE/sPAP ≤ 0.55 mm/mmHg, LVEF ≥ 65%) may identify a high-risk subgroup that could benefit from early comprehensive assessment, including systematic nutritional evaluation and closer follow-up after discharge.

The coexistence of impaired nutritional status and cardiopulmonary dysfunction may identify a particularly high-risk clinical phenotype. Catabolic and inflammatory states associated with malnutrition and infection may contribute to worsening pulmonary vascular function and increased RV afterload, thereby favoring the development of RV–PA uncoupling, while also influencing left ventricular structure and performance.

**Figure 3:**
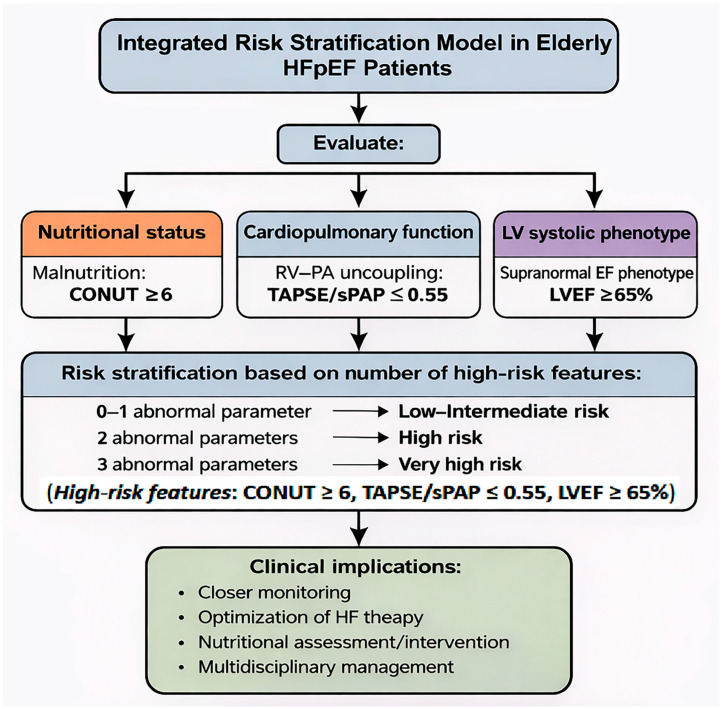
Integrated risk stratification model for elderly patients with heart failure with preserved ejection fraction (HFpEF) based on immunonutritional and cardiopulmonary parameters. The model is based on the combined assessment of three key domains: nutritional status (CONUT score), cardiopulmonary function (TAPSE/sPAP ratio), and left ventricular systolic phenotype (LVEF). High-risk features were defined according to cut-off values derived from ROC analysis (CONUT ≥ 6, TAPSE/sPAP ≤ 0.55 mm/mmHg, and LVEF ≥ 65%). Patients were stratified according to the number of abnormal parameters (0–1, 2, or 3), corresponding to increasing levels of risk (low–intermediate, high, and very high risk, respectively). The accumulation of these features reflects progressive biological and hemodynamic vulnerability and may support clinical decision-making, including closer monitoring, optimization of heart failure therapy, and targeted nutritional interventions. Cut-off values were derived from this cohort and require external validation. CONUT, Controlling Nutritional Status score; HFpEF, heart failure with preserved ejection fraction; LVEF, left ventricular ejection fraction; RV–PA, right ventricular–pulmonary arterial; sPAP, systolic pulmonary artery pressure; TAPSE, tricuspid annular plane systolic excursion.

To improve the clinical applicability of our findings, we propose an integrated risk stratification model based on the combined assessment of immunonutritional status, cardiopulmonary function, and left ventricular systolic phenotype (Figure 3). This simplified approach may facilitate the identification of particularly vulnerable HFpEF patients in routine clinical practice, although external validation is required.

Consistent with this pathophysiological framework, patients who died during follow-up more frequently exhibited a cardiac phenotype characterized by smaller LV cavity dimensions, increased wall thickness, and relatively hyperdynamic systolic function, consistent with concentric remodeling and supra-normal ejection fraction. However, these findings should be interpreted with caution, as higher LVEF in this clinical context may not represent a direct adverse mechanistic factor but rather a marker of a broader and more complex hemodynamic and clinical condition.

Previous studies have described a U-shaped relationship between ejection fraction and mortality in both general cardiovascular populations [82,83] and HF cohorts [77,78,79,80]. Nevertheless, in elderly and acutely ill patients, supranormal LVEF (≥65%) may reflect underlying pathophysiological states such as reduced ventricular cavity size, altered preload conditions, or increased sympathetic activation, rather than intrinsically enhanced systolic performance.

The mechanisms underlying HFsnEF are likely multifactorial and may include increased ventricular stiffness, reduced cavity size, elevated arterial elastance, and diastolic dysfunction, leading to impaired ventricular filling and reduced effective stroke volume despite elevated LVEF [84,85]. In our cohort, this pattern was frequently observed in the setting of acute systemic conditions, particularly infections, where factors such as dehydration, inflammation, and hemodynamic instability may further influence ventricular performance and loading conditions.

Taken together, these findings suggest that supra-normal LVEF should be interpreted as part of a complex clinical phenotype rather than as an isolated prognostic determinant. The coexistence of malnutrition, systemic inflammation, infection, and RV–PA uncoupling may identify a particularly vulnerable subgroup of HFpEF patients, underscoring the need for an integrated and multidimensional clinical assessment.

### 4.4. Strengths and Limitations

The present study has several strengths. First, it included a relatively large cohort of elderly patients with HFpEF, a population that remains underrepresented in many HF studies despite the increasing prevalence of HFpEF in advanced age. Second, the study provides long-term follow-up data, allowing a comprehensive evaluation of survival and clinical events over several years after hospitalization. Third, the analysis simultaneously evaluated multiple immunonutritional indices, enabling a direct comparison of their prognostic performance within the same patient population. Finally, the integration of clinical, laboratory, and echocardiographic variables, including indices of RV–PA coupling, allowed a multidimensional assessment of factors associated with prognosis.

However, several limitations should be acknowledged. The retrospective, single-center design may limit the generalizability of the findings and introduces the possibility of selection bias. Moreover, the absence of external validation in an independent cohort represents an important limitation; therefore, the reproducibility and broader applicability of these results should be confirmed in larger, multicenter prospective studies. In addition, no internal validation procedures (such as bootstrapping or cross-validation) were performed, and therefore the discriminative performance observed in ROC analyses may be subject to overestimation, while the identified cut-off values should be considered exploratory and require confirmation in independent cohorts.

Although a comprehensive set of clinical, laboratory, and echocardiographic variables was included, residual confounding due to unmeasured factors cannot be excluded. In particular, given the advanced age and high comorbidity burden of the study population, the potential influence of acute non-cardiac conditions—such as infections and respiratory diseases—on both immunonutritional indices and clinical outcomes cannot be fully excluded, and may have contributed to the observed associations.

The partial overlap among the immunonutritional indices evaluated may also introduce a degree of collinearity; however, this was mitigated by the analytical approach, which avoided the simultaneous inclusion of closely related variables in multivariate models.

Furthermore, the study population consisted of very elderly patients with HFpEF, which may limit the applicability of the findings to younger populations or to patients with different HF phenotypes.

Immunonutritional indices were assessed only at hospital admission, without longitudinal evaluation over time; thus, dynamic changes in nutritional and inflammatory status, which may carry additional prognostic value, were not captured.

Finally, patient enrollment took place in 2020, during the COVID-19 pandemic, a period that may have influenced both clinical presentation and outcomes due to changes in infection rates, healthcare system burden, and management strategies. Accordingly, the potential impact of this context should be considered when interpreting the results.

Overall, these limitations should be considered when interpreting the results, although they do not detract from the relevance of the findings regarding the prognostic role of nutritional status and cardiopulmonary functional parameters in elderly patients with HFpEF.

## 5. Conclusions

In this cohort of very elderly patients hospitalized with HFpEF and followed over a prolonged period, both nutritional status and cardiopulmonary functional parameters emerged as key determinants of long-term prognosis. Among the immunonutritional indices evaluated, the CONUT score demonstrated the strongest independent association with mortality, suggesting that combined alterations in nutritional and immune status play a relevant role in outcome stratification in this population.

In addition, echocardiographic parameters reflecting cardiopulmonary interaction, particularly the TAPSE/sPAP ratio, together with LVEF, provided complementary prognostic information related to RV–PA coupling and ventricular functional reserve.

Taken together, these findings highlight the potential clinical value of integrating simple nutritional indices with echocardiographic assessment of cardiopulmonary coupling in the risk stratification of elderly patients with HFpEF. A multidimensional approach combining systemic and cardiovascular markers may help identify particularly vulnerable phenotypes within this heterogeneous syndrome.

Future prospective studies are needed to confirm these observations and to determine whether targeted nutritional strategies and interventions aimed at improving cardiopulmonary function may translate into better outcomes in this high-risk population.

## Figures and Tables

**Table 1:** Reference ranges and clinical interpretation of immunonutritional indices used in the study.

Index	Calculation	Normal/Low Risk	Mild Nutritional Risk	Moderate–Severe Nutritional Risk
**PNI**	10 × albumin (g/dL) + 0.005 × lymphocyte count (/mm^3^)	≥45–50	40–45	<40
**CONUT Score**	Based on albumin, total cholesterol, and lymphocyte count	0–1	2–4	≥5
**CALLy Index**	(Albumin × lymphocyte count)/CRP	Higher values indicate better nutritional and inflammatory status; values ≥ 2–3 generally associated with better prognosis	<2–3 indicates increased risk	Lower values associated with worse prognosis

Thresholds reported in the literature may vary slightly depending on the study population and clinical context. In general, higher CONUT scores and lower PNI or CALLy values reflect more severe nutritional impairment and systemic inflammatory burden. CALLy, C-reactive protein–albumin–lymphocyte index; CONUT, Controlling Nutritional Status score; CRP, C-reactive protein; PNI, Prognostic Nutritional Index.

**Table 2:** Baseline clinical characteristics of elderly patients with HFpEF according to survival status.

	All Patients (n = 200)	Dead (n = 123)	Alive (n = 77)	*p* Value
**Demographics**				
Age (years)	86.6 ± 6.5	87.0 ± 6.6	86.0 ± 6.3	0.29
Females (%)	140 (70.0)	84 (68.3)	56 (72.7)	0.50
**Cardiovascular risk factors**				
Hypertension (%)	151 (75.5)	91 (74.0)	60 (77.9)	0.49
Smoking (%)	31 (15.5)	18 (14.6)	13 (16.9)	0.65
Diabetes (%)	57 (28.5)	38 (30.9)	19 (24.7)	0.36
Dyslipidemia (%)	99 (49.5)	60 (48.8)	39 (50.6)	0.80
Obesity (%)	25 (12.5)	15 (12.2)	10 (13.0)	0.87
**Noncardiovascular comorbidities**				
CKD (%)	109 (54.5)	79 (64.2)	30 (39.0)	**<0.001**
COPD (%)	42 (21.0)	26 (21.1)	16 (20.8)	0.96
Hypothyroidism (%)	37 (18.5)	25 (20.3)	12 (15.6)	0.39
Cognitive impairment (%)	82 (41.0)	60 (48.8)	22 (28.6)	**0.007**
**Cardiovascular comorbidities**				
History of CAD (%)	39 (19.5)	28 (22.8)	11 (14.3)	0.16
Previous TIA/stroke (%)	37 (18.5)	27 (22.0)	10 (13.0)	0.13
PAD (%)	48 (24.0)	34 (27.6)	14 (18.2)	0.15
**Physical examination**				
Dyspnea	100 (50.0)	60 (48.8)	40 (51.9)	0.68
Leg swelling	34 (17.0)	22 (17.9)	12 (15.6)	0.65
SBP (mmHg)	135 (120–150)	130.8 ± 31.0	140 (120–160)	**0.02**
DBP (mmHg)	70 (60–80)	69.3 ± 15.1	70 (60–80)	0.18
HR (bpm)	78.5 ± 17.2	80.9 ± 13.9	74.6 ± 13.3	**0.01**
Body temperature ≥ 37.5° (%)	78 (39.0)	63 (51.2)	15 (19.5)	**<0.001**

Continuous variables are presented as mean ± standard deviation or median (interquartile range), as appropriate. Categorical variables are presented as number (percentage). Comparisons were performed between patients who died during follow-up and those who were alive at the end of follow-up. Statistically significant *p*-values are shown in bold. CAD, coronary artery disease; CKD, chronic kidney disease; COPD, chronic obstructive pulmonary disease; DBP, diastolic blood pressure; HR, heart rate; PAD, peripheral artery disease; SBP, systolic blood pressure; TIA, transient ischemic attack.

**Table 3:** Laboratory parameters and nutritional risk scores according to survival status.

	All Patients (n = 200)	Dead (n = 123)	Alive (n = 77)	*p* Value
**Blood tests**				
Hemoglobin (g/dL)	11.1 (9.0–12.8)	11.1 (9.2–12.1)	11.1 (9.7–13.2)	0.37
WBCs (×10^9^/L)	9.35 (6.85–13.02)	10.62 (7.34–14.08)	9.12 (6.64–12.45)	**<0.001**
Neutrophils (×10^9^/L)	7.94 (4.81–11.63)	8.69 (5.92–14.44)	6.49 (3.91–9.79)	**<0.001**
Lymphocytes (×10^9^/L)	1.10 (0.79–1.59)	0.94 (0.69–1.19)	1.40 (0.92–2.01)	**0.01**
Platelets (×10^9^/L)	232 (164–297)	232 (164–291)	234 (174–299)	0.59
Glucose (mg/dL)	121 (97–155)	125 (99–157)	109 (97–135)	0.92
Iron (µg/dL)	45 (25–65)	40 (22–66)	50 (33–73)	**0.003**
Creatinine (mg/dL)	1.23 (0.87–2.05)	1.39 (0.92–2.21)	1.06 (0.65–1.83)	**0.02**
eGFR (mL/min/m^2^)	39 (23–60)	38 (23.5–60)	46 (29–82)	**0.006**
Sodium (mEq/L)	140 (136–144)	140 (135.3–144.0)	138 (134–142)	**0.03**
Potassium (mEq/L)	4.15 ± 0.71	4.15 ± 0.65	3.96 (3.57–4.54)	0.92
Calcium (mmol/L)	2.10 (1.14–2.30)	2.10 (1.34–2.20)	2.20 (2.10–2.30)	0.16
Total bilirubin (mg/dL)	0.70 (0.40–1.10)	0.60 (0.40–0.90)	0.70 (0.40–1.10)	0.15
Albumin (g/dL)	2.99 (2.57–3.31)	2.79 (2.42–3.16)	3.36 (3.05–3.66)	**<0.001**
Uric acid (mg/dL)	7.30 (5.40–9.80)	7.60 (5.55–9.60)	6.70 (4.90–8.30)	0.14
Total cholesterol (mg/dL)	139.5 (119.8–165.2)	134 (110–160)	150 (132–174)	**<0.001**
HDL cholesterol (mg/dL)	37.0 (27.0–45.0)	34 (24–42)	40 (32–55)	**<0.001**
LDL cholesterol (mg/dL)	77.0 (60.9–98.8)	73.4 (51–90.8)	92 (75–118)	**<0.001**
Triglycerides (mg/dL)	107 (86–147)	107 (85.5–146)	113 (89–147)	0.11
TSH (mIU/L)	1.39 (0.82–2.00)	1.39 (0.82–2.00)	1.29 (0.64–2.05)	0.45
CRP (mg/dL)	6.20 (1.20–15.80)	6.50 (2.90–15.36)	2.95 (0.80–7.50)	**<0.001**
NT-proBNP (pg/mL)	1566.5 (345.0–5055.5)	1750 (396–7331)	1017 (173–3596)	**<0.001**
hs-cTn (ng/mL)	40.0 (20.0–84.5)	40 (20–140)	40 (10–80)	0.27
**Nutritional risk scores**				
PNI	35.9 (31.6–39.7)	33.3 (29.6–36.8)	43.7 (36.6–44.7)	**<0.001**
CONUT score	7 (5–9)	8 (7–9)	4 (2–5)	**<0.001**
CALLy index	0.063 (0.015–0.199)	0.035 (0.014–0.075)	0.22 (0.09–0.46)	**<0.001**

Continuous variables are presented as mean ± standard deviation or median (interquartile range), as appropriate. Comparisons were performed between patients who died during follow-up and those who were alive at the end of follow-up. Statistically significant results are shown in bold. CALLy, C-reactive protein–albumin–lymphocyte index; CONUT, controlling nutritional status score; CRP, C-reactive protein; eGFR, estimated glomerular filtration rate; HDL, high-density lipoprotein; hs-cTn, high-sensitivity cardiac troponin; LDL, low-density lipoprotein; NT-proBNP, N-terminal pro-B-type natriuretic peptide; PNI, prognostic nutritional index; TSH, thyroid-stimulating hormone; WBC, white blood cells.

**Table 4:** Chest X-ray, electrocardiographic, and echocardiographic findings according to survival status.

	All Patients (n = 200)	Dead (n = 123)	Alive (n = 77)	*p* Value
**Chest X-ray findings**				
Normal radiographic pattern (%)	42 (21.0)	14 (11.4)	28 (36.4)	**<0.001**
Pulmonary congestion (%)	118 (59.0)	74 (60.2)	44 (57.1)	0.66
Radiographic evidence of pneumonia (%)	40 (20.0)	35 (28.4)	5 (6.5)	**<0.001**
**ECG findings**				
Sinus rhythm (%)	141 (70.5)	84 (68.3)	57 (74)	0.39
AF (%)	59 (29.5)	39 (31.7)	20 (26)	0.39
Normal IV conduction (%)	88 (44.0)	50 (40.7)	38 (49.4)	0.21
LAFB (%)	54 (27.0)	32 (26)	22 (28.6)	0.68
LBBB (%)	8 (4.0)	4 (3.3)	4 (5.2)	0.57
RBBB (%)	50 (25.0)	37 (30)	13 (16.9)	**0.04**
**Conventional TTE parameters**				
IVS (mm)	13 (12–15)	14.0 (12.0–16.0)	13 (12–15)	0.77
PW (mm)	10 (9–11)	10.0 (9.0–11.0)	10 (9–11)	0.55
LVEDD (mm)	42 (39–47)	41.0 ± 6.5	44 (39–48)	**0.001**
RWT	0.50 (0.44–0.55)	0.50 ± 0.09	0.47 ± 0.07	**0.01**
LVEDV (mL)	60 (45–88)	55.0 (44.0–75.0)	66 (50–95)	**0.02**
LVESV (mL)	20 (15–28)	17.6 (14.4–22.8)	25.0 (19.3–34.0)	**<0.001**
LVEF (%)	65 (60–70)	68.0 ± 4.8	60 (55–65)	**<0.001**
E/A	0.77 (0.66–1.55)	0.82 (0.66–1.55)	0.70 (0.60–1.25)	0.08
E/e’	16 (11–23)	17.0 (11.0–23.0)	16.0 (11.0–21.0)	0.30
LA A-P diameter (mm)	46 (41–52)	47.0 (41.0–53.0)	46 (40–53)	0.83
LA longitudinal diameter (mm)	56 (50–63)	57.0 (50.0–64.0)	56 (50–65)	0.82
LAV (mL)	77 (55–110)	85 (55–110)	75 (57–110)	0.54
More than mild MR (%)	63 (31.5)	38 (30.9)	25 (32.5)	0.82
More than mild AR (%)	8 (4.0)	3 (2.4)	5 (6.5)	0.13
More than mild AS (%)	23 (11.5)	13 (10.6)	10 (13)	0.63
More than mild TR (%)	57 (28.5)	36 (29.3)	21 (27.3)	0.77
RVIT (mm)	30 (26–35)	30.0 (26.0–35.0)	31 (27–35)	0.28
TAPSE (mm)	18 (14–22)	16 (11–28)	22 (20–25)	**<0.001**
sPAP (mmHg)	45 (25–100)	49 (24–100)	40 (25–100)	**<0.001**
TAPSE/sPAP (mm/mmHg)	0.45 (0.31–0.63)	0.32 (0.25–0.45)	0.74 (0.63–0.88)	**<0.001**
Aortic root (mm)	34.1 ± 4.1	33.0 ± 4.7	34.5 ± 4.6	0.13
Ascending aorta (mm)	35.3 ± 4.6	35.0 ± 4.6	36.0 ± 4.7	0.14

Continuous variables are presented as mean ± standard deviation or median (interquartile range), as appropriate. Categorical variables are presented as number (percentage). Comparisons were performed between patients who died during follow-up and those who were alive at the end of follow-up. Significant differences are indicated in bold. AF, atrial fibrillation; AR, aortic regurgitation; AS, aortic stenosis; ECG, electrocardiogram; IVS, interventricular septum; LA, left atrium; LAFB, left anterior fascicular block; LAV, left atrial volume; LBBB, left bundle branch block; LVEDD, left ventricular end-diastolic diameter; LVEDV, left ventricular end-diastolic volume; LVEF, left ventricular ejection fraction; LVESV, left ventricular end-systolic volume; MR, mitral regurgitation; PW, posterior wall; RBBB, right bundle branch block; RVIT, right ventricular inflow tract; RWT, relative wall thickness; sPAP, systolic pulmonary artery pressure; TAPSE, tricuspid annular plane systolic excursion; TR, tricuspid regurgitation; TTE, transthoracic echocardiography.

**Table 5:** Clinical presentation at admission, underlying etiology of heart failure, causes of hospitalization, and in-hospital treatment according to survival status.

	All Patients (n = 200)	Dead (n = 123)	Alive (n = 77)	*p* Value
**NYHA functional status at admission**				
NYHA III (%)	124 (62)	68 (55.3)	56 (72.7)	**0.01**
NYHA IV (%)	76 (38)	55 (44.7)	21 (27.3)	**0.01**
**Underlying etiology of heart failure**				
CAD (%)	64 (32)	44 (35.8)	20 (26)	0.14
VHD (%)	42 (21)	26 (21.1)	16 (20.8)	0.95
HHD (%)	60 (30)	30 (24.4)	30 (39)	**0.03**
PH (%)	34 (17)	30 (24.4)	4 (5.2)	**0.001**
**Main causes leading to hospital admission**				
CHF (%)	48 (24)	10 (8.1)	38 (49.3)	**<0.001**
Acute respiratory conditions (%)	87 (43.5)	68 (55.3)	19 (24.7)	**<0.001**
Gastro-intestinal disorders (%)	8 (4)	6 (4.9)	2 (2.6)	0.42
Severe anemia (%)	16 (8)	11 (8.9)	5 (6.5)	0.44
Severe CKD (%)	9 (4.5)	5 (4.1)	4 (5.2)	0.72
Cancers (%)	8 (4)	5 (4.1)	3 (3.9)	0.95
Hyponatremia (%)	16 (8)	12 (9.8)	4 (5.2)	0.22
Hypernatremia (%)	5 (2.5)	4 (3.3)	1 (1.3)	0.36
Neurological disorders (%)	3 (1.5)	2 (1.6)	1 (1.3)	0.86
≥2 causes of admission (%)	58 (29)	45 (36.6)	13 (16.9)	**0.002**
**Pharmacological therapy during hospitalization**				
Antiplatelets (%)	74 (37)	47 (38.2)	27 (35.1)	0.64
Anticoagulants (%)	82 (41)	50 (40.7)	32 (41.6)	0.89
ACEIs/ARBs (%)	84 (42.0)	40 (32.5)	44 (57.1)	**<0.001**
CCB (%)	51 (25.5)	29 (23.6)	22 (28.6)	0.46
BB (%)	115 (57.5)	68 (55.3)	47 (61)	0.43
Digoxin (%)	59 (29.5)	39 (31.7)	20 (26)	0.36
Loop diuretics (%)	132 (66)	82 (66.7)	50 (64.9)	0.77
MRAs (%)	73 (36.5)	47 (38.2)	26 (33.8)	0.55
Statins (%)	58 (29)	28 (22.8)	30 (39.0)	**0.02**
Corticosteroids (%)	58 (29)	41 (33.3)	17 (22.1)	0.08
Antibiotics (%)	126 (63)	101 (82.1)	25 (32.5)	**<0.001**
Oxygen therapy (%)	73 (36.5)	53 (43.1)	20 (26)	**0.02**
Oral hypoglycemic agents (%)	20 (10)	10 (8.1)	10 (13)	0.28
Insulin (%)	25 (12.5)	18 (14.6)	7 (9.1)	0.29
**LOS (days)**	9.4 ± 6.2	10.6 ± 6.5	7.5 ± 5.3	**0.01**

Continuous variables are presented as mean ± standard deviation, whereas categorical variables are presented as number (percentage). Comparisons were performed between patients who died during follow-up and those who were alive at the end of follow-up. Statistically significant values are shown in bold. ACEIs, angiotensin-converting enzyme inhibitors; ARBs, angiotensin receptor blockers; BB, beta-blockers; CAD, coronary artery disease; CCB, calcium channel blockers; CHF, congestive heart failure; CKD, chronic kidney disease; HHD, hypertensive heart disease; LOS, length of stay; MRAs, mineralocorticoid receptor antagonists; NYHA, New York Heart Association; PH, pulmonary hypertension; VHD, valvular heart disease.

**Table 6:** Mortality distribution and non-fatal clinical events during follow-up.

Category	n (%)
Overall mortality	123 (61.5)
Patients alive at end of follow-up	77 (38.5)
**Causes of death (n = 123)**	
Respiratory infections/respiratory failure	48 (39.0)
Cardiovascular causes (HF progression, acute cardiac events)	31 (25.2)
Multisystem deterioration/frailty-related conditions	21 (17.1)
Other non-cardiovascular causes	23 (18.7)
**Non-fatal events among survivors (n = 77)**	
Respiratory events (dyspnea, infections, COPD exacerbation)	30 (39.0)
Cardiovascular events (HF worsening, ACS)	14 (18.2)
Non-respiratory infections	10 (13.0)
Renal/metabolic complications	9 (11.7)
Trauma/falls/fractures	8 (10.4)
Other medical causes	6 (7.8)

Values are presented as number (percentage). Causes of death are reported among patients who died during follow-up (n = 123). Non-fatal events are reported among patients who were alive at the end of follow-up (n = 77). Percentages for causes of death and non-fatal events are calculated within the corresponding subgroup. ACS, acute coronary syndrome; COPD, chronic obstructive pulmonary disease; HF, heart failure.

**Table 7:** Univariate and multivariate Cox regression analysis for predictors of all-cause mortality.

Variable	Univariate HR (95% CI)	*p* Value	Multivariate HR (95% CI)	*p* Value
Age	1.00 (0.97–1.03)	0.755	—	—
Female sex	0.70 (0.48–1.03)	0.077	—	—
Diabetes mellitus	1.37 (0.93–2.02)	0.111	—	—
Coronary artery disease	1.21 (0.79–1.84)	0.377	—	—
Systolic blood pressure	0.997 (0.992–1.003)	0.324	—	—
eGFR	0.995 (0.986–1.004)	0.225	—	—
Sodium	1.002 (0.979–1.026)	0.872	—	—
Loop diuretics	1.20 (0.81–1.77)	0.354	—	—
E/e’	1.01 (0.99–1.04)	0.366	—	—
PNI	0.935 (0.909–0.961)	**<0.001**	1.001 (0.967–1.036)	0.940
CONUT score	1.244 (1.153–1.343)	**<0.001**	1.136 (1.027–1.256)	**0.013**
CALLy index	0.96 (0.69–1.34)	0.829	—	—
LVEF	1.093 (1.055–1.132)	**<0.001**	1.056 (1.014–1.099)	**0.008**
TAPSE/sPAP ratio	0.086 (0.035–0.213)	**<0.001**	0.222 (0.082–0.603)	**0.003**

Hazard ratios (HR) with corresponding 95% confidence intervals (CI) were calculated using Cox proportional hazards regression analysis. Variables significantly associated with mortality in the univariate analysis were entered into the multivariate model to identify independent predictors of all-cause mortality during follow-up. Statistically significant values are in bold. CALLy, C-reactive protein–albumin–lymphocyte index; CI, confidence interval; CONUT, controlling nutritional status score; eGFR, estimated glomerular filtration rate; HR, hazard ratio; LVEF, left ventricular ejection fraction; PNI, prognostic nutritional index; TAPSE, tricuspid annular plane systolic excursion; sPAP, systolic pulmonary artery pressure.

## Data Availability

The datasets generated and analyzed during the current study are publicly available on Zenodo at https://zenodo.org.
